# Biocomposite Materials Derived from *Andropogon halepensis*: Eco-Design and Biophysical Evaluation

**DOI:** 10.3390/ma17051225

**Published:** 2024-03-06

**Authors:** Marcela-Elisabeta Barbinta-Patrascu, Cornelia Nichita, Bogdan Bita, Stefan Antohe

**Affiliations:** 1 Department of Electricity, Solid-State Physics and Biophysics, Faculty of Physics, University of Bucharest, 405 Atomistilor Street, 077125 Magurele, Romania; marcela.barbinta@unibuc.ro (M.-E.B.-P.); bogdan.bita@fizica.unibuc.ro (B.B.); 2 CTT-3Nano-SAE Research Center, Faculty of Physics, ICUB, University of Bucharest, MG-38, 405 Atomistilor Street, 077125 Magurele, Romania; 3 National Institute for Chemical-Pharmaceutical Research and Development, 112 Vitan Avenue, 031299 Bucharest, Romania; 4 National Institute for Lasers, Plasma and Radiation Physics, Magurele, 077125 Bucharest, Romania; 5 Academy of Romanian Scientists, Ilfov Street 3, 050045 Bucharest, Romania

**Keywords:** silver nanoparticles, “green” synthesis, *Andropogon halepensis*, biocomposites, antioxidant activity, spider silk, *Pholcus phalangioides*

## Abstract

This research work presents a “green” strategy of weed valorization for developing silver nanoparticles (AgNPs) with promising interesting applications. Two types of AgNPs were phyto-synthesized using an aqueous leaf extract of the weed *Andropogon halepensis* L. Phyto-manufacturing of AgNPs was achieved by two bio-reactions, in which the volume ratio of (phyto-extract)/(silver salt solution) was varied. The size and physical stability of *Andropogon*—AgNPs were evaluated by means of DLS and zeta potential measurements, respectively. The phyto-developed nanoparticles presented good free radicals-scavenging properties (investigated via a chemiluminescence technique) and also urease inhibitory activity (evaluated using the conductometric method). *Andropogon*—AgNPs could be promising candidates for various bio-applications, such as acting as an antioxidant coating for the development of multifunctional materials. Thus, the *Andropogon*-derived samples were used to treat spider silk from the spider *Pholcus phalangioides*, and then, the obtained “green” materials were characterized by spectral (UV-Vis absorption, FTIR ATR, and EDX) and morphological (SEM) analyses. These results could be exploited to design novel bioactive materials with applications in the biomedical field.

## 1. Introduction

Nanotechnology, a cutting-edge multidisciplinary field in science and technology, has been penetrating more and more into fields such as cosmetics, medicine, or agriculture. In recent years, special attention has been paid to the development of new “green” nano-strategies to design materials with unusual properties by combining bio-organic and inorganic matter. Thus, current trends in nanotechnology are the use of bio-methods based on *Green Chemistry* principles (involving nontoxic precursors and mild reaction conditions) and the usage of bioresources, including bacteria, fungi, viruses, yeasts, algae, and vegetal extracts, that act as nano-factories for the “green” preparation of nanoparticles (NPs) [1]. Harnessing the huge potential of plants and plant waste for the “green” synthesis of metal nanoparticles is a current trend in nanotechnology. Recent reports demonstrated that leaf extracts are the best option for the biosynthesis of silver nanoparticles (AgNPs) due to the presence of phyto-compounds such as polyphenols, flavonoids, terpenoids, aliphatic alcohols, and aldehydes, which act both as bioreducing and capping agents in AgNPs synthesis. This process, called AgNPs phyto-synthesis, is based on *Green Chemistry* principles; therefore, it is a nontoxic, low-cost, and harmless process that uses natural resources, avoiding the use of hazardous ingredients [2,3]. Phyto-fabricated AgNPs (phyto-AgNPs) possess interesting bioactivities (e.g., antioxidant, antimicrobial, and antiproliferative qualities, among others) that make them suitable for biomedical applications [2,4,5]. Composite materials containing phyto-AgNPs have been applied as coatings for medical devices, medical fabrics, or scaffolds, diabetic socks, wound dressings, and tissue engineering and regeneration [2]. Biocomposites based on biocompatible polymers and phyto-AgNPs have antibacterial properties and may help protect a scaffold against infection [6].

The present work aimed to design “green” biocomposites based on a natural polymeric material, spider silk, and silver nanoparticles phyto-generated from an *Andropogon halepensis* aqueous extract. The development of these composites consisted of the following steps: (i) the preparation of the *Andropogon halepensis* aqueous extract from fresh leaves, (ii) the phyto-synthesis of AgNPs through two bio-reactions, (iii) the treatment of spider silk with the phyto-nanometallic particles, and (iv) the biophysical-chemical characterization of the *Andropogon*-derived materials.

Through this study, we wanted to show the huge value of natural resources, whether they are of vegetable (weeds) or insect origin (spider webs), in developing valuable hybrid materials for various applications. In this way, the use of “green” technologies and bio-waste will contribute to our planet’s safety, preserving “green” trees, “blue” waters, and blue sky.

*Andropogon halepensis* L., commonly known as Johnsongrass, is a warm-season-perennial grass, native to the Mediterranean region, which belongs to the Poaceae family [7]. This plant is known as one of the most common and troublesome weeds, with a worldwide distribution [8], meaning that it is an abundant free natural source which could be used both as a bioreducing and capping agent to develop metallic nanoparticles. The chemical composition of *Andropogon halepensis* L. can vary depending on its growth stage, environmental conditions, and geographic location. Its main chemical components are carbohydrates (represented by cellulose, hemicellulose, and sugars), proteins, lipids, and various phytochemicals, including phenolic compounds and flavonoids. The specific phenolic compounds found in *Andropogon halepensis* L. are ferulic acid, caffeic acid, p-coumaric acid, quercetin, and tannins [9,10,11,12]. Huang et al. reported for the first time, in 2010, the presence of three flavonoids—apigenin, tricin, and luteolin—in the aerial parts of this plant [13]. Also, the grass *Andropogon halepensis* L. contains essential minerals such as potassium, magnesium, and calcium and trace elements like iron, zinc, manganese, chromium, and copper [6]. *Andropogon halepensis* possesses various biological properties, among which we can mention its invasive character, adaptability to different types of soil, and drought resistance [14,15,16]. Regarding its pharmaceutical properties, the studies carried out by Shah et al. (2021) found antioxidant, antimicrobial, and antidiabetic properties in the methanolic extract obtained from the rhizomes of *A. halepensis* L. [12].

Thus, an important, *key point* in our study is to demonstrate that invasive plants can be converted into materials (AgNPs) beneficial for human health. Another *key point* in this work is the development of new materials through the functionalization of a natural biomaterial—that is, spider silk—with *Andropogon*-derived AgNPs.

Spider silk is a valuable bionic material in nature, with fascinating properties which are more performant than most artificial materials [17,18].

Spiders produce cobwebs to catch their prey, thus securing their food. Also, spiders use their webs for shelter, and they deposit their eggs in them. With the help of their cobwebs, spiders can move from one place to another and also “fly”, in a process called “ballooning”, during which spiders move through the air thanks to the electric fields detected by their electroreceptors [19].

Spider silk is an antimicrobial, hypoallergenic, and completely biodegradable natural proteic material [18]. Spider webs have been used since ancient times, such as by the Greeks and the Romans, who stopped battle wounds from bleeding by covering them with spider silk [20]. Spider webs are still used nowadays as a hemostatic agent and in wound healing [21]. Moreover, spider silk is also used as a bioactive material for tissue engineering and drug delivery due to its excellent biocompatibility and biodegradability [22].

As spider silk has as its main component a protein (spidroin), it is biocompatible, nontoxic, and fully biodegradable [23]. Spider silk is an ideal material for wound suture and prosthesis since human tissues can absorb its degradation products.

In addition, spider silk has been used in the textile field to make clothes that are similar to worm silk but have a better performance in terms of being lightweight, non-breakable, with good breathability, strong water absorption, and UV resistance qualities. Despite these interesting features, the production efficiency of spider silk is low, and spider silk clothing is a luxury [23,24] because spider webs must be collected from many spiders, and, in addition, spiders are cannibals and should not be grown in close proximity.

Moreover, spider silk–metallic nanoparticles composites have been developed and used for biomedical applications, including as antimicrobial and anticoagulant agents [25].

As it is known, biocomposites combine the properties of all their components. In this regard, taking into account the properties of phyto-AgNPs and spider webs, the current study proposes the functionalization of spider webs (arising from the spider *Pholcus phalangioides*) with phyto-AgNPs obtained from *Andropogon halepensis*, as mentioned above.

To our knowledge, there is no prior report on the use of the weed *Andropogon halepensis* to “green” develop biocomposites with spider silk. In this paper, the obtained biocomposites were biophysico-chemically characterized, and their potential use in the biomedical field is discussed.

## 2. Materials and Methods

### 2.1. Materials

Rutin trihydrate (≥94.0%), gallic acid (≥97.5%), Folin–Ciocalteu’s phenol reagent, sodium carbonate anhydrous (≥99.0%), hydrochloric acid (HCl, 37%), aluminum chloride (99.99%), silver nitrate (AgNO_3_), urea (99.5%), sodium acetate (≥99.0%), luminol (5-amino-2,3-dihydro-phthalazine-1,4-dione), dimethyl sulfoxide (DMSO, ≥99.9%), hydrogen peroxide (H_2_O_2_, 30%), hydroxy methyl aminomethane base (TRIS ≥ 99.8%), and methanol (≥99.9%) were purchased from Merck Company (Darmstadt, Germany). Urease from Jack Bean was acquired from Fisher Scientific (Oxford, UK), and the conductivity standard solution utilized (1413 µS/cm) was purchased from Hanna Instruments. For the preparation of the vegetal aqueous extract, fresh leaves of *Andropogon halepensis* L. (Johnsongrass) were harvested on June 2022, from the Prahova county (Romania) (44°50′03″ N 25°53′57″ E).

### 2.2. Preparation of Andropogon-Derived Materials

#### 2.2.1. Preparation of the Phyto-Extract

Fresh leaves of *Andropogon halepensis* were washed many times with tap water to remove any dust and then rinsed in distilled water. The cleaned leaves were further chopped into small pieces, immersed in boiling distilled water, and then boiled for 15 min. The mass ratio of *plant leaves*/*distilled water* reached a value of 1:4. The obtained phyto-extract (referred to as EAh) was filtered through Whatman filter paper no. 1 and kept in the freezer until use.

#### 2.2.2. Biological Nano-Synthesis of Silver Nanoparticles

In this study, two types of silver nanoparticles were nano-synthesized, using the prepared phyto-extract, through the following bioreducing reactions:

*Bio-Reaction 1*: A volume of 1 mL of the prepared *A. halepensis* extract was mixed with 1 mL of 1 mM AgNO_3_ aqueous solution, in the dark, under continuous magnetic stirring (VIBRAX stirrer, Milian, OH, USA, 200 rpm), at room temperature. After 24 h, the color stabilized as a dark-brown-green color, indicating the completion of the phyto-synthesis of the AgNPs, a fact which was further demonstrated by UV-Vis absorption spectroscopy. The obtained AgNPs were named AgNPs_1.

*Bio-Reaction 2*: A volume of 1 mL of the prepared phyto-extract was mixed with 100 mL of 1 mM AgNO_3_ aqueous solution, in the dark, under continuous magnetic stirring (VIBRAX stirrer, Milian, OH, USA, 200 rpm), at room temperature. After 45 min, the color of the mixture changed. After 24 h, the color stabilized as a reddish brown, indicating the completion of NPs synthesis, a fact which was further proved by UV-Vis absorption spectroscopy. These AgNPs were named AgNPs_2.

The phyto-molecules from the *Andropogon halepensis* extract gave up electrons to silver ions (arising from the AgNO_3_ solution), reducing and then surrounding them, forming nanoparticles. Thus, the EAh acted both as the bioreducing and capping agent for the silver ions.

The biosynthesized AgNPs samples were also monitored after 18 months. The “new” synthesized nanoparticles were referred to as AgNPs_1n and AgNPs_2n, and the “old” ones were referred to as AgNPs_1o and AgNPs_2o.

#### 2.2.3. Preparation of Biocomposites Based on Silk and Phyto-Extract/Phyto-Nanometals

**Figure 1:**
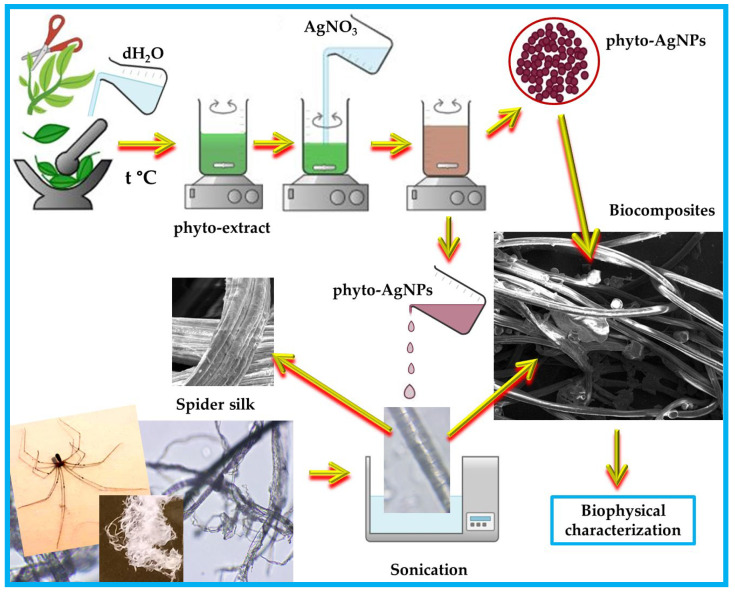
Schematic representation of the eco-design and preparation of the materials derived from *Andropogon halepensis* L. The figure was created with Chemix (https://chemix.org/, accessed on 25 January 2024) and with PowerPoint and Paint 3D. This figure also contains images taken by us with a camera, an optical microscope, and SEM.

The spider cobwebs used in these experiments were harvested, using a long stick, from the spider *Pholcus phalangioides* (common name: “Daddy” Long Legs [26]) (from Bucharest, Romania), which is a common Romanian spider with extremely long legs (Figure 1). The cobwebs were washed in a mild detergent, then rinsed several times with distilled water, and left to dry at room temperature.

The spider web samples were divided into many experimental batches: (1) untreated (sample S), (2) treated with EAh (sample S_EAh), (3) treated with AgNPs_1 (sample S_AgNPs_1), and (4) treated with AgNPs_2 (sample S_AgNPs_2).

The codes of the spider web samples studied are presented in Table 1. The treatment was performed by ultrasound irradiation in a water bath (BRANSON 1210, Marshall Scientific, Hampton, NH, USA) for 60 min (with a break after 30 min). Then, the spider webs were left for another 5 h at room temperature in the suspensions in which they had been immersed, then they were removed from the suspensions and left to dry, and then they were analyzed by SEM/EDS, UV-Vis, and FTIR-ATR spectroscopy.

Table 1 displays the abbreviations of all the samples obtained during this research.

The schematic representation of the eco-design of all the samples is shown in Figure 1.

### 2.3. Physical-Chemical Characterization of Andropogon-Derived Materials

A JASCO UV-Vis V-570 spectrophotometer (Jasco International Co., Ltd., Tokyo, Japan) was used to record the **UV-Vis absorption** spectra of the phyto-extract and silver nanoparticles in the 200–800 nm wavelength and at a scanning rate of 1 nm/s, at 25 °C. The UV-Vis absorbance spectra of the spider silk-based materials were recorded on the JASCO UV–Vis 530 spectrophotometer with an integrating sphere accessory.

**The chromatic parameters** of the pristine and treated silk samples were measured on the same equipment using the JASCO’s color diagnosis system (CIE LAB system of colors).

The following parameters were calculated: L*, a*, and b*, using the CIE LAB system of colors. The three coordinates of the CIE LAB system represent the following:

○ L* represents the lightness (L* = 0 indicates black, L* = 100 indicates white);
○ a* indicates the relative position between green and red (negative values of a* indicate a green color, and positive values indicate a red color);
○ b* represents the relative position between blue and yellow (negative values of b* indicate the color blue, and positive values indicate the color yellow).

Based on the different values in the color parameters between the reference (untreated spider silk) and the sample (treated spider silk), the color difference (ΔE*) of the sample from the reference was calculated using the following equation [27,28]:

∆E* = [(∆L*)^2^ + (∆a*)^2^ + (∆b*)^2^]^1/2^ (1)

**Fourier-transform IR (FTIR)** spectra were collected using a Perkin Elmer Spectrum 400 instrument with an **attenuated total reflectance (ATR)** diamond crystal, in a transmittance mode. Scans in the 4000–650 cm^−1^ range were accumulated for each spectrum at a spectral resolution of 4 cm^−1^.

Further investigations into the morphostructural aspects of the sample were conducted using the Apreo S ThermoFisher scanning electron microscope (**SEM**), with a field emission gun (FEG), operating at 10 kV in a secondary electron mode, equipped with a 9 µm aperture and a sample-to-detector distance of 9 mm. To facilitate imaging, the samples were affixed to a conductive self-adhesive carbon tape and securely positioned on the aluminum SEM holder cap. In the case of liquid samples, a drop-casting method was employed for deposition. Elemental analysis was performed using energy dispersive X-ray spectroscopy (**EDX**) with the EDAX TEAM system, using a 10 kV excitation voltage and a 70 mm^2^ area detector to gain valuable insights into the samples’ composition and structural characteristics.

The average size of particles (Zav) was determined, in triplicate, by **dynamic light scattering (DLS)**, and the results were reported as the mean values ± S.D. **DLS measurements** were performed on a Zetasizer Nano ZS (Malvern Instruments Inc., Worcestershire, UK), at a temperature of 25 °C, using 173° backscatter angle detection, assuming a laser beam at a wavelength of 633 nm. The polydispersity index, PdI, as the indicator of the size distribution’s width, was also determined.

**Zeta potential** (ξ, mV) measurements were performed in triplicate, at 25 °C, in an appropriate device, the Zetasizer Nano ZS (Malvern Instruments Ltd., Malvern, UK), by applying an electric field across the analyzed samples. Zeta potential measurements were carried out in ultrapure water, at a pH of 6.98. The ξ values were reported as mean ± S.D.

### 2.4. Bio-Evaluation of the Andropogon-Derived Samples

#### 2.4.1. Total Polyphenol Content (TPC)

The **total polyphenol content (TPC)** of the vegetal extract was determined by means of the UV-Vis spectrophotometric method using the Folin–Ciocalteu assay [29,30]. In this assay, the Folin–Ciocalteu reagent, a mixture of phosphotungstic (H_3_PW_12_O_40_) and phosphomolybdic (H_3_PMo_12_O_40_) acids, is generally reduced to blue oxides of tungsten (W_8_O_23_) and molybdenum (Mo_8_O_23_) during phenol oxidation. This reaction takes place in alkaline conditions provided by a sodium carbonate solution. Briefly, in our experiment, a volume of 100 µL of vegetal extract was mixed with 0.5 mL of Folin–Ciocalteu phenol reagent (Merck, Darmstadt, Germany), followed by the addition of 2 mL of an anhydrous sodium carbonate (Na_2_CO_3_ 99% purity, Merck Company) solution (20%). The above-mentioned mixture was incubated for 60 min, and the optical absorbance was recorded at 760 nm using a JASCO UV-Vis V-570 spectrophotometer (Jasco International Co., Ltd., Tokyo, Japan). The TPC values were calculated using a gallic acid calibration curve, and the results were expressed as mg gallic acid (≥97.5% purity, Merck Company) equivalent/g dry extract (mg GAE g^−1^) [31,32]. All the experiments were replicated three times.

#### 2.4.2. Total Flavonoid Content (TFC)

**The total flavonoid content (TFC)** of the vegetal extract was estimated using an aluminum chloride colorimetric assay, as previously described [33]. Briefly, an aliquot of 5 mL of the *A. halepensis* extract or of AgNPs was mixed with 5.0 mL of sodium acetate (≥99.0% purity, Merck Company) 100 g/L, 3.0 mL of AlCl_3_ (99.99% purity, Merck Company) 25 g/L, and filled-up to 25 mL with methanol (≥99.9%, purity, Merck Company) in a volumetric flask. After incubation at room temperature for 30 min, the optical absorbances of the reaction mixtures were recorded at 430 nm using the JASCO UV-Vis V-570 spectrophotometer (Jasco International Co., Ltd., Tokyo, Japan). The TFC was determined using a rutin calibration curve, and the results were expressed as mg rutin trihydrate (≥94.0% purity, Merck Company) equivalent/g dry extract (mg RE g^−1^). All the experiments were performed in triplicate.

#### 2.4.3. In Vitro Antioxidant Activity Assay

**The in vitro non cellular antioxidant activity** of the *Andropogon halepensis* extract and its phyto-derived nanoparticles was evaluated by means of the chemiluminescence (CL) technique, using the procedure described elsewhere [3,34]. The CL experiments were carried out on a Sirius Luminometer Berthold–GmbH (Pforzheim, Germany), using luminol-H_2_O_2_ as a generator system (in TRIS-HCl pH 8.65) for reactive oxygen species. The value of the in vitro antioxidant activity (%AA) for each sample was calculated using the following equation:

(2) $$AA(\%)=\frac{I_{0}-I_{S}}{I_{0}}\times 100$$

where I_0_ = CL intensity for control (the reaction mixture without sample) at t = 5 s; and I_s_ = CL intensity in the presence of the tested sample, at t = 5 s. For each sample, the %AA results were reported as the mean values of three CL assays.

#### 2.4.4. Investigation of Urease Inhibitory Activity of Andropogon-Derived AgNPs

The urease-inhibiting activity of *Andropogon* metallic nanoparticles was evaluated by measuring electrical conductivity (Cobra3 Chem-Unit, Phywe System GmbH, Göttingen, Germany), as described in [34,35]. Urease (urea amidohydrolase, EC 3.5.1.5) is the enzyme that catalyzes the hydrolysis of urea into ammonia and carbon dioxide; the ions arising from this reaction’s products increase a medium’s conductivity. The resulting conductivity value is closely correlated with the rate of urea hydrolysis reaction. Therefore, the reaction rate can be evaluated as the variation in time of conductivity. A volume of 50 μL of urease solution [in 50% glycerine (1000 U/mL)] was added to 40 mL of 1.6% urea solution, under continuous stirring. Then, after 300 s, a volume of 1 mL of the sample was added into this reaction mixture. The electrical conductivity evolution was monitored on the PC using the measureAPP software (https://www.phywe.com/sensors-software/measurement-software-apps/measureapp-the-free-measurement-software-for-all-devices-and-operating-systems_2274_3205/, accessed on 1 April 2023).

## 3. Results and Discussion

### 3.1. Evaluation of Total Phenolic and Total Flavonoid Contents of Andropogon halepensis Extract

Phenolic compounds are bioactive phyto-molecules that have grafted one or multiple hydroxyl groups on an aromatic ring and are responsible for scavenging free radicals, which explains their antioxidant properties. Flavonoids are one type of phenolic compounds with a wide range of bioactivities.

The TPC of the Andropogon halepensis aqueous leaf extract, determined using the Folin–Ciocalteu method, was 20.83 ± 0.96 mg GAE/g dry extract. Shah et al. had previously reported a TPC value of 20.3 ± 1.5 mg GAE/g dry extract for the aerial parts of *S. halepense* aqueous extract [36], and Mohammed et al. [37] reported a polyphenol content of 20.78 ± 1.83 mg GAE/g dry extract for an aqueous extract of aerial parts of Beta vulgaris.

The TFC value of the *Andropogon halepensis* aqueous leaf extract was TFC = 9.12 ± 0.62 mg RE/g dry extract, a value which is comparable with the results for other extracts produced [38].

### 3.2. Optical Characterization of Andropogon-Derived Materials

The samples were optically characterized by UV-Vis absorption spectra and also by FTIR ATR spectroscopy. Moreover, chromatic characterization was carried out.

**Figure 2:**
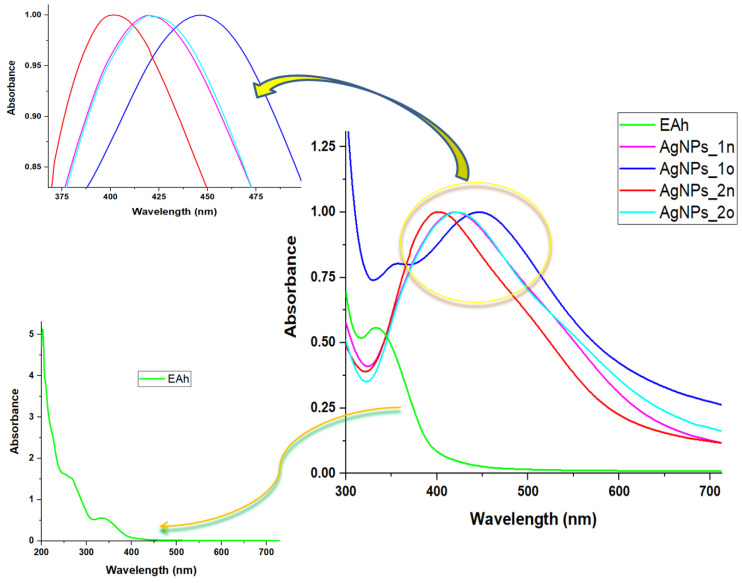
Comparative presentation of UV-Vis absorption spectra of *Andropogon*-derived samples. All AgNPs spectra were normalized at their maximum. The top inset shows the SPR bands of the *Andropogon*-derived AgNPs. The bottom inset shows the spectrum of *Andropogon halepensis* aqueous extract (EAh).

The UV-Vis absorption spectra (Figure 2) of *A. halepensis*-derived silver nanoparticles showed a single strong peak for each type of AgNPs, located at 402 nm, 419 nm, 423 nm, and 447 nm for AgNPs_2n, AgNPs_1n, AgNPs_2o, and AgNPs_1o, respectively. In this wavelength range of 400–500 nm, the vegetal extract did not show any peaks. These findings are consistent with those found in previous works [39,40,41]. The above-mentioned maxima were only due to metallic NPs formation and are generally called SPR (surface plasmon Resonance) bands, which are produced by the electromagnetic field which induces the collective oscillation of the electrons in the conduction band on the nanoparticles’ surface [42]. According to the Mie theory [43], the presence of only one SPR band indicates the formation of spherical AgNPs. The SPR peaks of our old samples broadened and shifted to higher wavelengths, due to their aggregation tendency, indicating an increase in AgNPs size. On the contrary, narrow peaks at shorter wavelengths indicated a decrease in AgNPs size [44]. Thus, the dimensions of AgNPs_1n and AgNPs_2o appeared to be very close. These assumptions were further confirmed by DLS, zeta potential measurements, and SEM analysis. In addition, the phyto-extract showed UV-Vis absorption bands characteristic for polyphenolic compounds (335 nm), and for carbohydrates, and the aromatic amino acid residues of proteins (247–270 nm) [34].

The chromatic parameters and the color change values (ΔE*) of the silk materials are displayed in Table 2. For the silk samples treated with *A. halepensis* extract and its derived AgNPs, the value of L* decreased compared to the pristine spider silk samples (S), indicating that the whiteness of S decreased after the treatments. The values of the chromatic parameters a* and b* of the spider silk samples have increased after treatment, indicating a slight shift to red and yellow, respectively. So, the color of the samples was in the yellow–greenish–green range. The color difference (ΔE*) had small values after the treatment of fresh *Andropogon*-derived AgNPs. Greater values of ΔE* were obtained for spider silk treated with 18 months-aged AgNPs (S_AgNPs_1o and S_AgNPs_2o).

*Andropogon*-derived AgNPs can be used in the treatment of spider silk–based fabrics/materials when the aim is not to change the color of the fibers but to preserve the color and only slightly intensify its tones. This can be an important aspect in their use in the conservation/treatment of the colored objects, including, for example, the treatment of heritage objects, as mentioned in [45].

**Figure 3:**
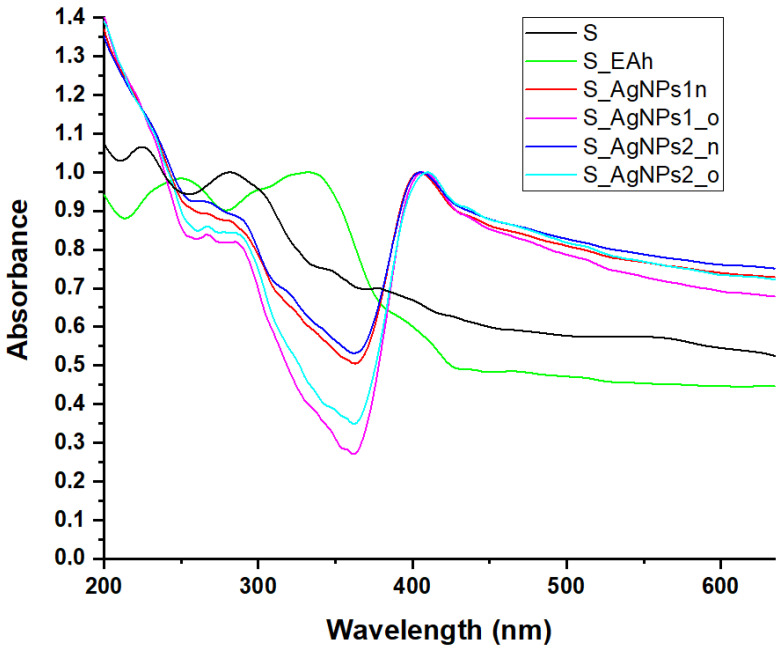
Comparative presentation of UV-Vis absorption spectra of the spider silk samples untreated (S) and treated with plant extract (S_EAh) or with silver nanoparticles obtained by *Bio-Reaction 1* (S_AgNPs_1n and S_AgNPs_1o) and *Bio-Reaction 2* (S_AgNPs_2n and S_AgNPs_2o).

The UV-Vis absorption spectra of the silk materials in our study presented the specific signatures of phyto-nanosilver in the case of the spider silk treated with silver nanoparticles (Figure 3). After treatment with the vegetal extract EAh, the spectrum of spider silk shifted to longer wavelengths. A bathochromic shift was also observed in the case of the spectra of the spider silk treated with aged silver nanoparticles compared to fresh ones.

**Figure 4:**
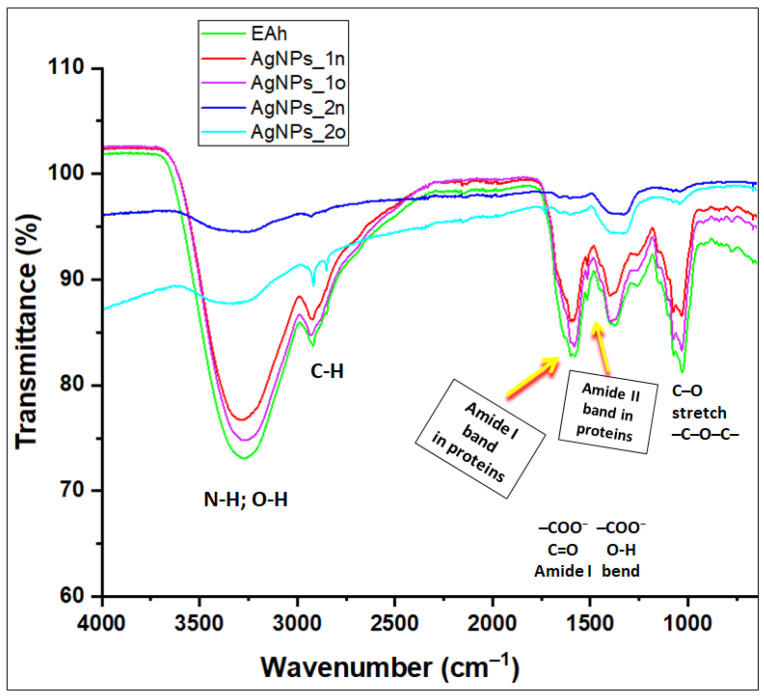
Comparative presentation of FTIR ATR spectra of *Andropogon halepensis* extract (EAh) and the derived AgNPs phyto-synthesized through *Bio-Reaction 1* (AgNPs_1n and AgNPs_1o) and *Bio-Reaction 2* (AgNPs_2n and AgNPs_2o). The index “n” refers to the “new” synthesized nanoparticles, while the index “o” refers to the “old” (18 months aged) ones.

**Figure 5:**
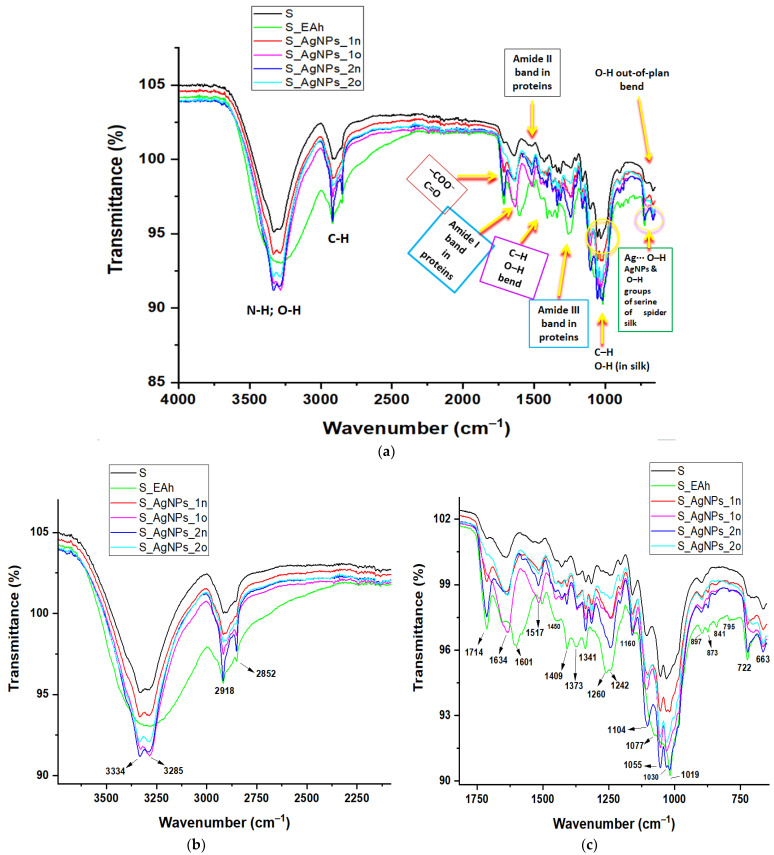
Comparative presentation of FTIR ATR spectra of the spider silk samples untreated (S) and treated with plant extract (S_EAh) or with silver nanoparticles obtained by *Bio-Reaction 1* (S_AgNPs_1n and S_AgNPs_1o) and *Bio-Reaction 2* (S_AgNPs_2n and S_AgNPs_2o) (**a**). Insets show the magnified regions of the FTIR ATR spectra of the biocomposites (**b**,**c**).

The *Andropogon*-derived samples were further investigated with FTIR ATR spectroscopy (Figure 4 and Figure 5). The attributions of the main FTIR bands are displayed in Tables S1 and S2 (Supplementary Material).

The FTIR ATR spectra provided useful information about the phyto-compounds (created from the leaf extract) wrapping the surface of nanoparticles (see Figure 4).

The intense band centered at 3277 cm^−1^ in the FTIR ATR spectrum of the phyto-extract (assigned to bending and stretching vibrations of hydroxyl groups intermolecularly hydrogen-bonded in phenolic compounds and polysaccharides and also to the stretching vibrations of the primary and secondary amines) shifted to 3286 cm^−1^ and 3319 cm^−1^ in the FTIR ATR spectra of AgNPs_1n and AgNPs_2n, respectively. These findings suggest that phyto-synthesized silver nanoparticles carry, on their metallic surface, amino and hydroxyl groups associated through hydrogen bonding.

The samples EAh, AgNPs_1n, and AgNPs_2n presented FTIR ATR bands attributed to a C–H anti-symmetric stretching vibration, located at wavenumbers 2920, 2925, and 2930 cm^−1^, respectively, and also the C–H symmetrical stretch vibration of alkyl chains located at wavenumbers 2845, 2851, and 2861 cm^−1^, respectively (see Table S1 in the Supplementary Material).

In the fingerprint region, the narrow and medium band at 1369 cm^−1^ (assigned to carboxylates; phenol or tertiary alcohol, O–H bend; primary or secondary, O–H in-plan bend; C–H bend) in the EAh spectrum was weakened after the addition of silver nitrate and shifted to 1398 cm^−1^ in the AgNPs_1n spectrum and to 1354 cm^−1^ in the AgNPs_2n spectrum. Moreover, this band broadened in the case of AgNPs_2n.

The peak at 1261 cm^−1^, ascribed to a primary or secondary O–H in-plan bend, aromatic ethers, and aryl–O stretching, was weakened after the bio-reaction in the case of AgNPs_1n and disappeared in the case of AgNPs_2n.

After the bioreducing reaction, the band at 1030 cm^−1^ observed in the spectrum of EAh (characteristic for the stretching mode of the –C–O group of polysaccharides and chlorophyll, the –C–O–C– group in ethers and secondary alcohols, and C–O bending in esters) shifted to 1074–1040 cm^−1^ in the AgNPs_1n spectrum. In addition, these bands were weakened in the nanoparticle spectrum.

The characteristic vibration band for amide I (due to carbonyl stretch in proteins) in the spectrum of the vegetal extract shifted towards longer wavenumbers after the addition of the silver nitrate solution. Moreover, this band weakened and broadened in the AgNPs_2n spectrum. In addition, the small peak assigned to amide II due to N–H bending and C–N stretching in proteins disappeared in the AgNPs_2n spectrum.

As observed, the shape of the FTIR ATR spectra of the silver nanoparticles obtained through bio-reaction (1)—AgNPs_1n and AgNPs_1o—closely resembled the spectrum of the phyto-extract EAh, while the FTIR ATR spectra of the silver nanoparticles obtained through bio-reaction (2)—AgNPs_2n and AgNPs_2o—were much different from those of the samples previously mentioned.

After 18 months, some spectral changes occurred in the spectrum of the silver nanoparticles.

FTIR ATR spectra confirmed the development of phyto-AgNPs. This analysis suggested the involvement of phyto-molecules originated from the *Andropogon halepensis* extract, especially polyphenols, flavonoids, proteins, carboxylic acids, polysaccharides, chlorophylls, esters, and ethers, in the bioreduction of silver ions and the development of AgNPs. The obtained silver nanoparticles carried on their surfaces various functional groups (e.g., hydroxyl, carbonyl, and amino) associated by hydrogen bonding.

A comparative presentation of the FTIR ATR spectra of the untreated and treated spider silk samples is given in Figure 5.

After treatment with the phyto-extract, the FTIR ATR spectrum of spider silk underwent important changes in the wavenumber region of 3500–3000 cm^−1^ (Figure 5b), indicating the role of hydroxyl groups (belonging to the phenolic compounds and flavonoids from the phyto-extract) in the surface coverage of the spider silk fibers. Moreover, in the fingerprint region (Figure 5c), there were many changes in the Amide I, II, and III bands in the spider silk proteins. In addition, more bands appeared in the region 897–722 cm^−1^ in the spectrum of S_EAh, indicating the presence of hydroxyl groups at the surface of the spider silk fibers.

Similar changes occurred in the same regions in the spectra of the spider silk treated with metallic nanoparticles.

It can be observed that there were changes in the spectra of spider silk treated with the “old” AgNPs compared to those of silk treated with the fresh ones (Figure 5a–c).

### 3.3. Evaluation of the Zeta Potential of the Phyto-Derived Metallic Nanoparticles

**Figure 6:**
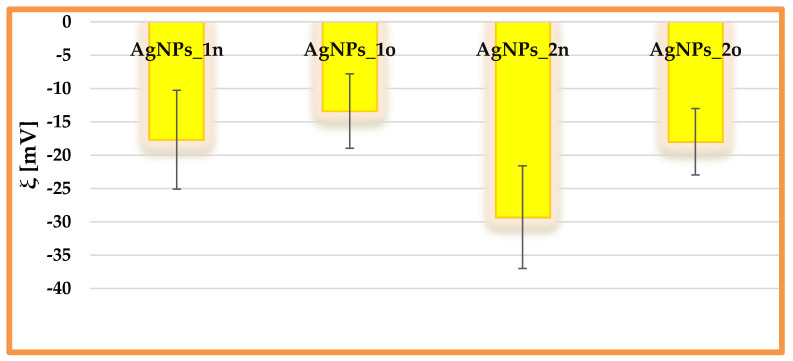
Comparative presentation of the electrokinetic potential of the *Andropogon*-derived silver nanoparticles. The AgNPs samples obtained through *Bio-Reaction* 1 (AgNPs_1n and AgNPs_1o) are placed next to those obtained through *Bio-Reaction* 2 (AgNPs_2n and AgNPs_2o) by alternating the aged samples with the fresh ones.

Zeta potential measurements revealed the negative charges of the phyto-nanometallic particles due to the presence of carboxylate and hydroxyl groups on their surfaces, arising from proteins and polyphenols, respectively (as demonstrated by UV-Vis and FTIR ATR spectroscopy). Figure 6 displays a comparative presentation of the zeta potential values for the *Andropogon*-derived AgNPs. The particles AgNPs_2 were more stable than AgNPs_1, since they had a more negative value for the zeta potential; therefore, electrostatic repulsion was more pronounced in this case. AgNPs_1n presented a moderate physical stability (ξ = −17.7 ± 7.41 mV), while AgNPs_2n showed a good stability (ξ = −29.3 ± 7.70 mV). These values obtained for our developed AgNPs are in line with other reports in the scientific literature. For instance, Vishwasrao et al. prepared AgNPs from sapota (*Manilkara zapota*) pomace extract, with a moderate stability (ξ = −13.41 ± 0.02 mV) [46]. Malaka et al. [47] synthesized AgNPs from *Zea mays* husk extract, with a zeta potential value of −28.7 mV.

After 18 months, the phyto-nanometallic particles had aggregated, a fact shown by the decrease in ξ magnitude.

### 3.4. Size, Morphological, and Compositional Characterization of Spider Silk Biocomposites and Their Building Blocks

**Figure 7:**
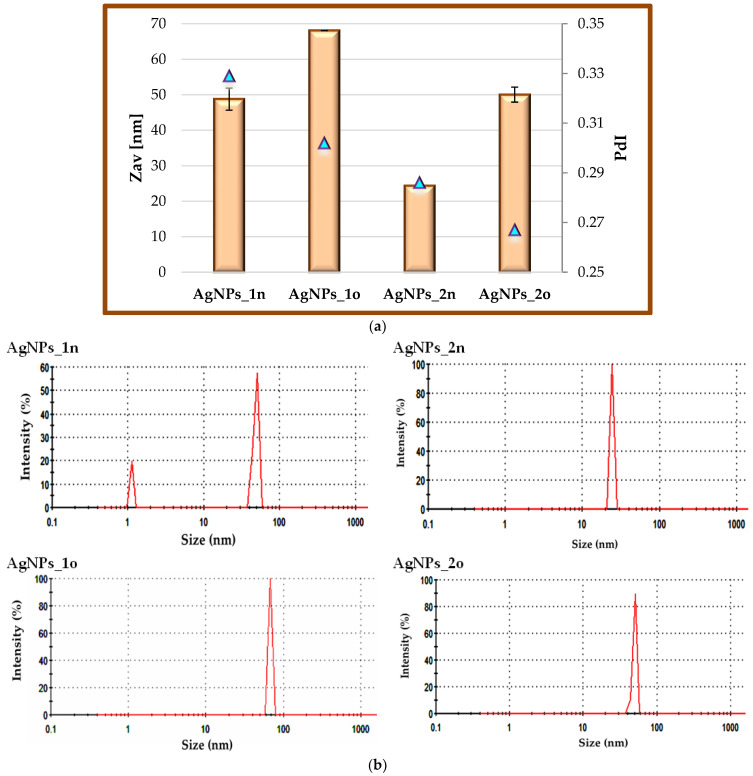
(**a**) Comparative presentation of the average particle size (Zav, nm) and PdI index of “green”-developed silver nanoparticles, estimated by dynamic light scattering (DLS) measurements; (**b**) Size distribution profiles of particle population for all types of phyto-developed AgNPs. For comparison, the aged AgNPs samples (AgNPs_1o and AgNPs_2o) are arranged next to the fresh ones (AgNPs_1n and AgNPs_2n).

The average particle size (Zav, nm) values of the *Andropogon*–derived nanoparticles and the polydispersity indices (PdI), estimated by dynamic light scattering (DLS) measurements, are displayed in Figure 7. The values of Zav were in the range 24–68 nm, with a PdI index between 0.267 and 0.329 (Figure 7a). The smallest size was recorded for AgNPs_2n (24.36 nm), which was also the most stable sample (see Section 3.3).

Figure 7b displays a narrow size distribution profile of the particle population for all the types of phyto-developed AgNPs. It can be seen that the size of both types of nanoparticles increased after 18 months.

The obtained results are within the range of AgNPs size reported for AgNPs synthesized from *Azadirachta Indica* L. leaves extract (20–50 nm) [48] and from aqueous extracts of aerial parts of *Astragalus spinosus* (10–60 nm) [49].

**Figure 8:**
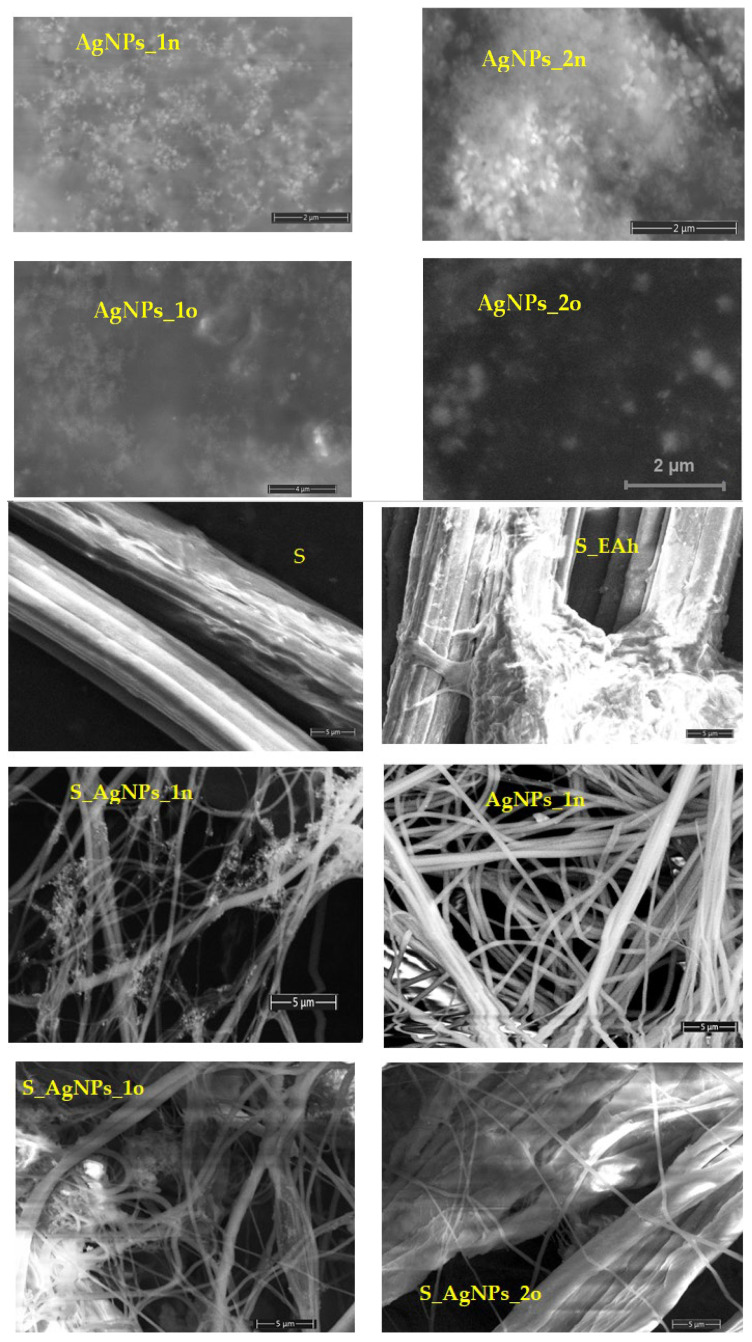
The SEM images of the phyto-developed AgNPs (AgNPs_1n, AgNPs_1o, AgNPs_2n, and AgNPs_2o) and of the spider silk fibers (S) and spider silk biocomposites with plant extract (S_EAh) or with silver nanoparticles (S_AgNPs_1n, S_AgNPs_1o, S_AgNPs_2n, and S_AgNPs_2o).

The morphological aspects of the samples were analyzed by SEM analysis (Figure 8). The SEM micrographs revealed spherical metallic nanoparticles (AgNPs_1 and AgNPs_2), confirming that the *A. halepensis* extract acted both as a bioreducing and capping agent in the development of the silver nanoparticles. The spherical shape of the phyto-developed AgNPs was predicted by the UV-Vis absorption spectra (see Section 3.2). Similar results were also reported by Sathishkumar et al. for the “green” synthesis of silver nanoparticles using *Morinda citrifolia* L. [50]. The SEM image of the sample S_EAh showed silk bundle fibers with a phyto-matrix. The silk treatment with the silver nanoparticles under ultrasound irradiation led to silk nanofibers functionalized with AgNPs. The SEM micrographs showed the presence of AgNPs on the surface of the treated silk nanofibers: S_AgNPs_1n, S_AgNPs_1o, S_AgNPs_2n, and S_AgNPs_2o. The functionalization of spider silk was more effective when freshly prepared AgNPs were used compared to aged ones (see samples S_AgNPs_1n and S_AgNPs_2n).

The EDX spectra (Figure S1, Supplementary Material) evidenced the presence of silver in the phyto-nanometallic samples (AgNPs_1n, AgNPs_1o, AgNPs_2n, and AgNPs_2o) and in the silk-biocomposites S_AgNPs_1n, S_AgNPs_1o, S_AgNPs_2n, and S_AgNPs_2o. Other chemical elements (O, Na, P, K, and Cl) came from the plant extract.

### 3.5. Investigation of Antioxidant Activity of Andropogon Extract and Its Derived AgNPs

**Figure 9:**
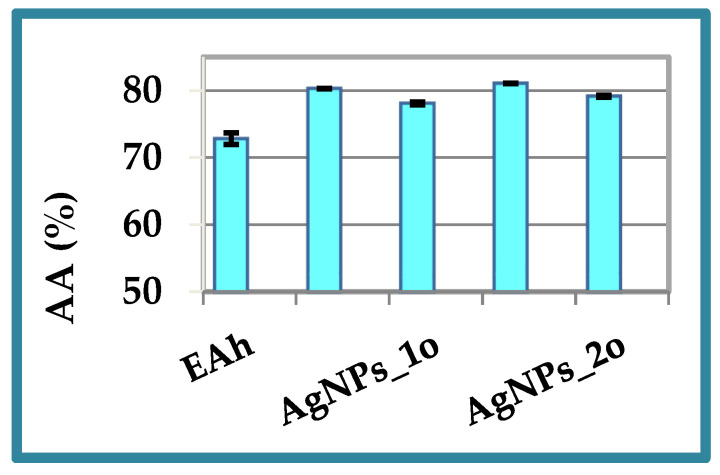
The antioxidant activity of the *Andropogon halepensis* extract (EAh) and the phyto-metallic particles obtained by *Bio-Reaction 1* (AgNPs_1n and AgNPs_1o) and *Bio-Reaction 2* (AgNPs_2n and AgNPs_2o), estimated using the chemiluminescence technique.

The in vitro antioxidant activity of the *A. halepensis* extract and the derived phyto-metallic particles was evaluated using the chemiluminescence technique. The AA% values varied in the range 74–81%, and the results are displayed in Figure 9. The ability of the *Andropogon* extract to scavenge free radicals was due to the presence of bioactive molecules such as polyphenols and flavonoids (see Section 3.1 and Section 3.2). The good antioxidant properties of the developed AgNPs were retained even after 18 months. Only slight changes in the antioxidant activity values occurred.

The antioxidant activity of the phyto-developed silver nanoparticles reached values greater than the activity of the precursor extract due to the presence, on their surface, of phyto-compounds (capping agents) from the vegetal extract and due to a nanosized effect that generates many reaction centers for the capturing of free radicals. This behavior of phyto-nanometallic particles compared to their phyto-extract precursors was highlighted in our previous studies [3,34]. In the current experiment, the best antioxidant properties were obtained for AgNPs_2n due to two key factors: (1) a good physical stability (see Figure 6) and (2) the smallest dimension (see Figure 7). The CL results were in good agreement with the DLS and zeta potential measurements.

### 3.6. Investigation of Effects of Andropogon Extract and Derived AgNPs on Urease Activity

The potential urease-inhibiting effect of the *Andropogon halepensis* extract and its developed phyto-metallic particles was estimated by means of the conductometric technique (Figure S2, Supplementary Material), which is a very simple approach.

Urea is an important organic molecule used in dermatology as a moisturizer and a keratolytic agent as well as in wound healing [51]. Urea is broken down by the enzyme urease into carbon dioxide and ammonia, resulting in the alkalization of the medium. Urease has been identified as a virulence factor for several microbial pathogens [52], being involved in many diseases’ pathogenesis in both humans and animals. Thus, finding novel urease inhibitors is an important research topic.

In the absence of a sample, during the first 300 s of the reaction evolution in our experiment (Figure S1, in Supplementary Material), the conductivity continuously increased due to the formation of ions during the hydrolysis reaction of urea as a result of the action of urease. After the addition of the samples, at t = 300 s, the conductivity drastically increased at this point due to the introduction of ions, originating from the sample, in the reaction medium. After this moment, if the conductivity increased, the sample either did not have any inhibitory actions (in the case of EAh) or had a slight inhibitory effect (see AgNPs_1n). In the case of sample AgNPs_2n, no further increase in conductivity was observed because the enzyme was inhibited and no transformation of the substrate (urea) was possible. The results shown in Figure S2 (Supplementary Material) reveal that the vegetal extract exhibited no inhibitory action on urease, while the phyto-derived AgNPs presented an inhibitory effect.

As seen in Figure S2 (Supplementary Material), AgNPs_1n showed a slight inhibitory action, while AgNPs_2n exhibited a strong inhibitory action against urease. Practically, urease lost its enzymatic activity in the presence of AgNPs_2n, and no further urea transformation was possible. This behavior could be explained by the smallest size of AgNPs_2n, allowing for a better interaction with urease’s active site, which could have changed urease’s conformation and then blocked this active site with nanoparticles [53].

The functionalization of spider silk with AgNPs_2n, which have an inhibitory effect on urease, could be exploited in the development of medical textiles that can be applied on skin treated with urea, thus preventing the latter’s decomposition.

## 4. Conclusions

This study presented a simple eco-design of biocomposites containing silk webs from spider *Pholcus phalangioides* and silver-based nanoparticles phyto-synthesized (using two phyto-reactions) from an aqueous extract of *Andropogon halepensis* leaves, a common weed. To the best of our knowledge, *Andropogon halepensis* has not been previously used for the development of such biocomposites.

Through this study, we highlighted the importance of using natural resources such as spider silk and plants, even invasive ones, to build biocomposites with potential applicability in the biomedical field.

The phyto-synthesis of the two types of AgNPs was demonstrated using spectral methods (UV-Vis and FTIR ATR) and by means of SEM analysis. The FTIR ATR investigation showed the involvement of organic molecules such as polyphenols, flavonoids, proteins, carboxylic acids, polysaccharides, chlorophylls, esters, and ethers, originating from the *Andropogon halepensis* extract, in the “green” synthesis of silver nanoparticles. The nanoscale dimension of the phyto-metallic particles was demonstrated by the DLS measurements and confirmed by the SEM images. Their zeta potential values indicated moderate-to-good physical stability. Certain changes in the properties of the obtained AgNPs were observed after 18 months, especially regarding their size and zeta potential values. On the contrary, their antioxidant properties did not undergo significant changes. The biophysical characterization of the phyto-metallic nanoparticles showed that sample AgNPs_2 (with the smallest ratio of phyto-extract/AgNO_3_) presented the best results, including in terms of its antioxidant properties and urease inhibitory activity.

The *Andropogon*-derived metallic nanoparticles were used to develop biocomposites with *Pholcus phalangioides* spider silk by means of a simple “green” bottom–up approach. The optical characterization (UV-Vis and FTIR ATR) and SEM analysis demonstrated the functionalization of spider silk with *Andropogon*-derived AgNPs. The FTIR ATR analysis suggested the key role of proteins, phenolic compounds, and flavonoids in the development of biocomposites.

The biophysical characterization of the phyto-metallic nanoparticles showed that the fresh AgNPs obtained via the second type of bio-reaction (sample AgNPs_2n) presented the best results in terms of physical stability, antioxidant activity, and urease-inhibiting activity. Moreover, this sample was the most efficient in the functionalization of spider silk. Thus, the spider silk-based composites developed in this study obtained by means of silk’s functionalization with AgNPs_2 could be further exploited in development of medical textiles that can be applied on skin treated with urea, thus preventing urea decomposition, and assuring the scavenging of free radicals. Moreover, our “green”-developed biocomposites can be applied also in various fields, such as biomedicine, environmental remediation, and nanotechnology, where these materials could potentially make significant contributions.

## Figures and Tables

**Table 1:** Names and description of the *Andropogon halepensis*-derived samples.

Sample Name	Description
**EAh**	Aqueous extract of *Andropogon halepensis* L. leaves
**AgNPs_1o**	Silver nanoparticles phyto-synthesized from *Andropogon halepensis* extract, using method 1 (the “old” sample)
**AgNPs_1n**	Silver nanoparticles phyto-synthesized from *Andropogon halepensis* extract, using method 1 (the “new” sample)
**AgNPs_2o**	Silver nanoparticles phyto-synthesized from *Andropogon halepensis* extract, using method 2 (the “old” sample)
**AgNPs_2n**	Silver nanoparticles phyto-synthesized from *Andropogon halepensis* extract, using method 2 (the “new” sample)
**S**	Spider silk (untreated) collected from the spider *Pholcus phalangioides*
**S_EAh**	Spider silk treated with *Andropogon halepensis* extract
**S_AgNPs_1o**	Spider silk treated with AgNPs_1o
**S_AgNPs_1n**	Spider silk treated with AgNPs_1n
**S_AgNPs_2o**	Spider silk treated with AgNPs_2o
**S_AgNPs_2n**	Spider silk treated with AgNPs_2n

**Table 2:** Chromatic parameters of untreated and treated spider silk samples.

Color Parameters				Spider Silk Sample		
	S	S_EAh	S_AgNPs_1n	S_AgNPs_1o	S_AgNPs_2n	S_AgNPs_2o
L*	98.05	97.74	97.00	88.77	96.72	91.15
a*	−6.97	−5.25	−5.21	−5.25	−5.19	−6.63
b*	5.53	7.63	5.75	5.76	5.75	6.95
ΔL*	-	−0.31	−1.05	−9.28	−1.33	−6.9
Δa*	-	1.72	1.76	1.72	1.78	0.34
Δb*	-	2.1	0.22	0.23	0.22	1.42
ΔE*	-	2.73	2.06	9.44	2.23	7.05

## Data Availability

The data were included in the text.

## Supplementary Materials

The following supporting information can be downloaded at https://www.mdpi.com/article/10.3390/ma17051225/s1: Table S1: FTIR ATR band assignment for vegetal extract and phyto-derived metallic nanoparticles; Table S2: FTIR ATR band assignment for untreated and treated spider silk; Figure S1: The EDX spectra of all the samples: *Andropogon halepensis* extract (EAh) and *Andropogon*-derived AgNPs (AgNPs_1n, AgNPs_1o, AgNPs_2n, and AgNPs_2o), spider silk (S), and spider silk composites with plant extract (S_EAh) or with silver nanoparticles (S_AgNPs_1n, S_AgNPs_1o, S_AgNPs_2n, and S_AgNPs_2o), at 10 kV; and Figure S2: Investigation of urease inhibitory action of the *Andropogon halepensis* extract and the developed phyto-metallic nanoparticles, estimated by the conductometric method. Refs. [54,55,56,57,58,59,60,61] are cited in the Supplementary Materials.
