# Supplementary material for: Biocomposite Materials Derived from Andropogon halepensis: Eco-Design and Biophysical Evaluation
Source: Materials (Basel). 2024 Mar 6;17(5):1225. doi: 10.3390/ma17051225 (PMC10934486; doi:10.3390/ma17051225)
Supplement: Supplementary file 1 [file materials-17-01225-s001.zip › materials-2889339-supplementary.pdf]

materials

MDPI

Supplementary material

Biocomposite Materials Derived from *Andropogon halepensis* – Eco-design and Biophysical Evaluation

M.-E. Barbinta-Patrascu[1], C. Nichita[2,3]*, B. Bita[1,4], S. Antohe[1,5]*

[1] Department of Electricity, Solid-State Physics and Biophysics, Faculty of Physics, University of Bucharest, 405 Atomistilor Street, 077125 Magurele, Romania; marcela.barbinta@unibuc.ro (M.-E.B.-P.); bogdan.bita@fizica.unibuc.ro (B.B.)
[2] CTT-3Nano-SAE Research Center, Faculty of Physics, ICUB, University of Bucharest, MG-38, 405 Atomistilor Street, 077125 Magurele, Romania
[3] National Institute for Chemical-Pharmaceutical Research and Development, 112 Vitan Avenue, 031299 Bucharest, Romania
[4] National Institute for Lasers, Plasma and Radiation Physics, Magurele, 77125 Bucharest, Romania
[5] Academy of Romanian Scientists, Ilfov Street 3, 050045 Bucharest, Romania
* Correspondence: santohe@solid.fizica.unibuc.ro or s_antohe@yahoo.com (S.A.); cornelia.nichita@unibuc.ro or cornelianichita@yahoo.com (C.N.)

Table S1. FTIR ATR band assignment for vegetal extract and phyto-derived metallic nanoparticles

Sample	FT-IR Bands (cm^{-1})	Assignment	Ref.
EAh	3277 (sharp, narrow, strong)	O–H bending and stretching vibrations of hydroxyl groups belonging to phenolic compounds and polysaccharides;<br>Stretching vibrations of the primary and secondary amines	[1]<br>[2]
	2920 (weak)	C–H anti-symmetric stretching vibration	[2]
	2845 (weak)	C–H symmetrical stretch vibration of alkyl chains	[2]
	1645 (shoulder),<br>1585 (strong)	Amide I, due to carbonyl stretch in proteins; Carboxylate groups (–COO^-); Stretching C=C (aromatic ring); Secondary amine, N–H bend	[1]<br>[2]
	1517 (weak, sharp)	Amide II due to N–H bending and C–N stretching in proteins	[3]<br>[4]
	1369 (medium, sharp)	Carboxylates; Phenol or tertiary alcohol, O–H bend; Primary or secondary, O–H in-plane bend; C–H bend	[2]
	1261 (weak)	Primary or secondary O–H in-plan bend; Aromatic ethers, aryl–O stretch; Aromatic primary amine, C–N stretch	[2]

	1030<br>(strong, sharp)	Cyclic ethers, large rings, C–O stretch; –C–O–C– ether<br>Antisymmetric stretching of –C–O group of polysaccharides and/or chlorophyll;<br>C–O bending in esters; Secondary alcohol, C–O stretch; Primary amine, C–N stretch	[1]<br>[2]
**AgNPs_1n/ AgNPs_1o**	3286/3270<br>(sharp, strong)	O–H stretching alcohols, polysaccharides and phenolic compounds/ polyphenols; Stretching vibrations of the primary and secondary amines	[1]<br>[2]
	2925/2930 (weak)	C–H anti-symmetric stretching vibration	[2]
	2851/2880<br>(shoulder)	C–H symmetrical stretch vibration of alkyl chains	[2]
	1591/1585<br>(strong, sharp)	Amide I, due to carbonyl stretch in proteins; Carboxylate groups ($–COO^–$);<br>Stretching C=C (aromatic ring); Primary amine, N–H bend	[1]<br>[2]
	1512/1517<br>(weak, sharp)	Amide II, due to N–H bending and C–N stretching in proteins	[3]<br>[4]
	1398/1394<br>(medium, sharp)	Carboxylates; Phenol or tertiary alcohol, O–H bend; Primary or secondary, O–H in-plane bend; C–H bend	[2]
	1261/1265<br>(shoulder)	Aromatic primary amine, C–N stretch;<br>Primary or secondary O–H in-plane bend; Aromatic ethers, aryl–O stretch	[2]
	1030/1035<br>(strong, sharp)	Cyclic ethers, large rings, C–O stretch; –C–O–C– ether<br>Antisymmetric stretching of –C–O group of polysaccharides and/or chlorophyll;<br>C–O bending in esters; Secondary alcohol, C–O stretch; Primary amine, C–N stretch	[1]<br>[2]
**AgNPs_2n/AgNPs_2o**	3319/ 3348<br>(very broad)	Very broad intense band overlapping the frequencies of the following groups: Hydrogen-bonded O–H (the bending and stretching vibrations of hydroxyl groups in alcohols, polysaccharides and phenolic compounds)	[1]<br>[2]
	2930/2920<br>(very weak, sharp)	C–H anti-symmetric stretching vibration; Transition metal carbonyls	[2]
	2861/2851<br>(very weak, sharp)	C–H symmetrical stretch vibration of alkyl chains	[2]
	1605/1605<br>(broad, very weak)	Amide I, arising due to carbonyl stretch in proteins; Carboxylate groups ($–COO^–$);<br>Stretching C=C (aromatic ring); Primary amine, N–H bend	[1]<br>[2]
	1354/1354<br>(medium, broad)	Carboxylates; Phenol or tertiary alcohol, O–H bend; Primary or secondary, O–H<br>in-plan bend; C–H bend	[2]
	1074, 1040/1079,<br>1044<br>(very weak)	Primary amine, C–N stretch; Alkyl-substituted ether; –C–O–C stretching vibration;<br>Primary alcohol, C–O stretch	[2]<br>[5]

Table S2. FTIR ATR band assignment for untreated and treated spider silk

Sample	FT-IR Bands (cm⁻¹)	Assignment	Ref.
S/ S_EAh	3332, 3286 (strong narrow band, splitted in 2 bands)/3281(broad)	Normal ''polymeric'' OH stretch; bending and stretching vibrations of hydroxyl groups intermolecularly hydrogen bonded, in alcohols, polysaccharides and phenolic compounds/ polyphenols; Stretching vibrations of the primary and secondary amines	[1] [2]
	2910 (broad, medium)/2918 (medium, sharp)	C–H anti-symmetric stretching vibration	[2]
	2854 (shoulder)/ 2852 (weak, sharp)	C–H symmetrical stretch vibration of alkyl chains	[2]
	1711 (very weak)/ 1714 (strong, sharp)	Carboxylates, C=O stretching	[6]
	1643 (medium, broad)/ 1655,1601 (medium)	Amide I, arising from carbonyl (–C=O) stretch in spider silk proteins	[3]
	1515, 1540/1517, 1503 (very weak)	N–H bending belonged to the Amide II regions of spider silk proteins	[3]
	1428 (weak, sharp)/ 1450 (weak, broad)	C–H bending	[2]
	1409, 1372, 1338, 1315 (weak, broad)/ 1409, 1373, 1341 (weak, sharp)	Phenol or tertiary alcohol, O–H bend ; C–H bend	[2]
	1278-1239 (very broad, weak)/1260, 1242 (medium)	Amide III band in proteins	[7]
	1161 (medium, sharp)/ 1155 (shoulder)	C–N stretch	[2]
	1106-1031 (sharp)/ 1077 (shoulder), 1019 (strong)	C–H and O–H bond vibrations because of the prevalence of hydroxyl amino acid side chains.	[3]
	898 (weak)/ 897-795 (weak bands), 722 (medium, sharp)	Hydrogen bonded O–H out-of-plan bending; Aromatic C–H out-of-plan bending	[2]
	665 (weak)/ 663 (weak, sharp)	C–H bend; Alcohol, O–H out-of-plan bend	[2]
S_AgNPs_1n/ S_AgNPs_1o	3332, 3281/3330, 3285 (strong narrow band, split-ted in 2 bands)/	Hydrogen-bonded O–H (the bending and stretching vibrations of hydroxyl groups in alcohols, polysaccharides and phenolic compounds); Stretching vibrations of the primary and secondary amines	[1] [2] [6]
	2918/ 2915 (medium, sharp)	C–H anti-symmetric stretching vibration	[2]
	2852 (shoulder)/ 2850 (weak,	C–H symmetrical stretch vibration of alkyl chains	[2]

	sharp)		
	1712 (medium, sharp)/ 1709 (shoulder)	Carboxylates, C=O stretching (in silk proteins)	[6]
	1640 (medium)/ 1634 (strong)	Amide I, due to carbonyl (–C=O) stretch in silk proteins and in proteins which cover AgNPs	[3] [8]
	1519 (weak, broad)/ 1517 (medium)	Amide II arising from N–H bending and C–N stretching in proteins	[1] [3] [4]
	1450, 1429/1452,1431 (weak bands)	C–H bending	[2]
	1407-1315/ 1409-1317 (weak)	Phenol or tertiary alcohol, O–H bend ; C–H bend	[2]
	1242/ 1242 (medium, broad)	Amide III band in proteins	[7]
	1161/1158 (medium, sharp)	C–N stretch	[2]
	1102-1018/ 1158-1028 (medium bands)	C–H and O–H bond vibrations because of the prevalence of hydroxyl amino acid side chains.	[3]
	895-793/ 897-845 (weak bands)	Hydrogen bonded O–H out-of-plan bending; Aromatic C–H out-of-plane bending	[2]
	722, 664/ 702, 662 (weak)	C–H bend; Alcohol, O–H out-of-plan bend; vibration of Ag···O bond between AgNPs and O–H groups of serine of spider silk proteins	[2] [9]
**S_AgNPs_2n/ S_AgNPs_2o**	3334, 3292/ 3337, 3291 (a splitted band, strong)	Very broad intense band overlapping the frequencies of the following groups: Phenols, O–H stretch; Hydrogen-bonded O–H (the bending and stretching vibrations of hydroxyl groups in alcohols, polysaccharides and phenolic compounds)	[1] [2]
	2918/2917 (medium, sharp)	C–H anti-symmetric stretching vibration	[2]
	2852 (medium, sharp)/ 2851 (very weak)	C–H symmetrical stretch vibration of alkyl chains	[2]
	1714 (strong, sharp)/1715 (very weak)	Carboxylates, C=O stretching (in silk proteins)	[6]
	1637 (broad, medium)/ 1635 (strong)	Amide I, arising from carbonyl (–C=O) stretch in silk proteins and in proteins which cover AgNPs	[3] [8]
	1517 (medium)/ 1518 (weak)	Amide II band due to N–H bending and C–N stretching in proteins	[1] [3] [4]

	1472 (shoulder), 1454 and 1427 (very weak)/ 1450 and 1432 (very weak)	C–H bending	[2]
	1409 (weak, sharp), 1370 (weak), 1338 (medium, sharp) and 1315 (weak, sharp)/ 1409, 1338 and 1316 (weak, sharp)	Phenol or tertiary alcohol, O–H bend ; C–H bend	[2]
	1245 (strong, sharp)/ 1245 (weak, broad)	Amide III band in proteins	[7]
	1160 / 1162 (medium, sharp)	C–N stretch	[2]
	1104-1018/ 1105-1029 (strong bands)	C–H and O–H bond vibrations because of the prevalence of hydroxyl amino acid side chains.	[3]
	898-793 (weak bands)/ 899 (weak), 873 (shoulder)	Hydrogen bonded O–H out-of-plan bending; Aromatic C–H out-of-plane bending	[2]
	722 (medium, sharp),663 (weak, sharp)/ 704 (very weak, broad), 665 (weak, sharp)	C–H bend; Alcohol, O–H out-of-plan bend; vibration of Ag···O bond between AgNPs and O–H groups of serine of spider silk proteins	[2] [9]

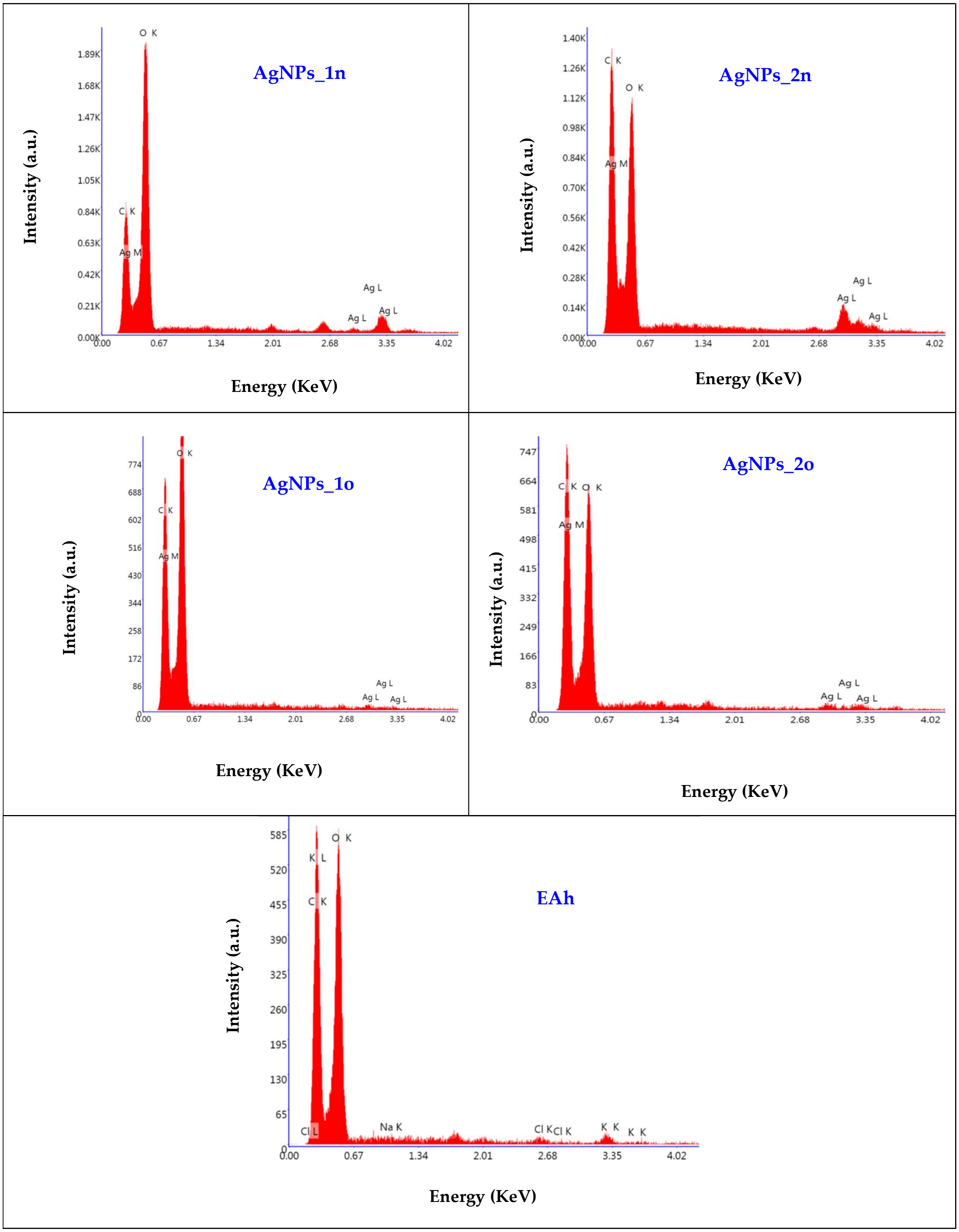
AgNPs_1n
O K
C K
Ag M
Ag L
Intensity (a.u.)
Energy (KeV)
AgNPs_2n
C K
O K
Ag M
Ag L
Intensity (a.u.)
Energy (KeV)
AgNPs_1o
O K
C K
Ag M
Ag L
Intensity (a.u.)
Energy (KeV)
AgNPs_2o
C K
O K
Ag M
Ag L
Intensity (a.u.)
Energy (KeV)
EAh
O K
K L
C K
Cl L
Na K
Cl K
Cl K
K K
K K
Intensity (a.u.)
Energy (KeV)

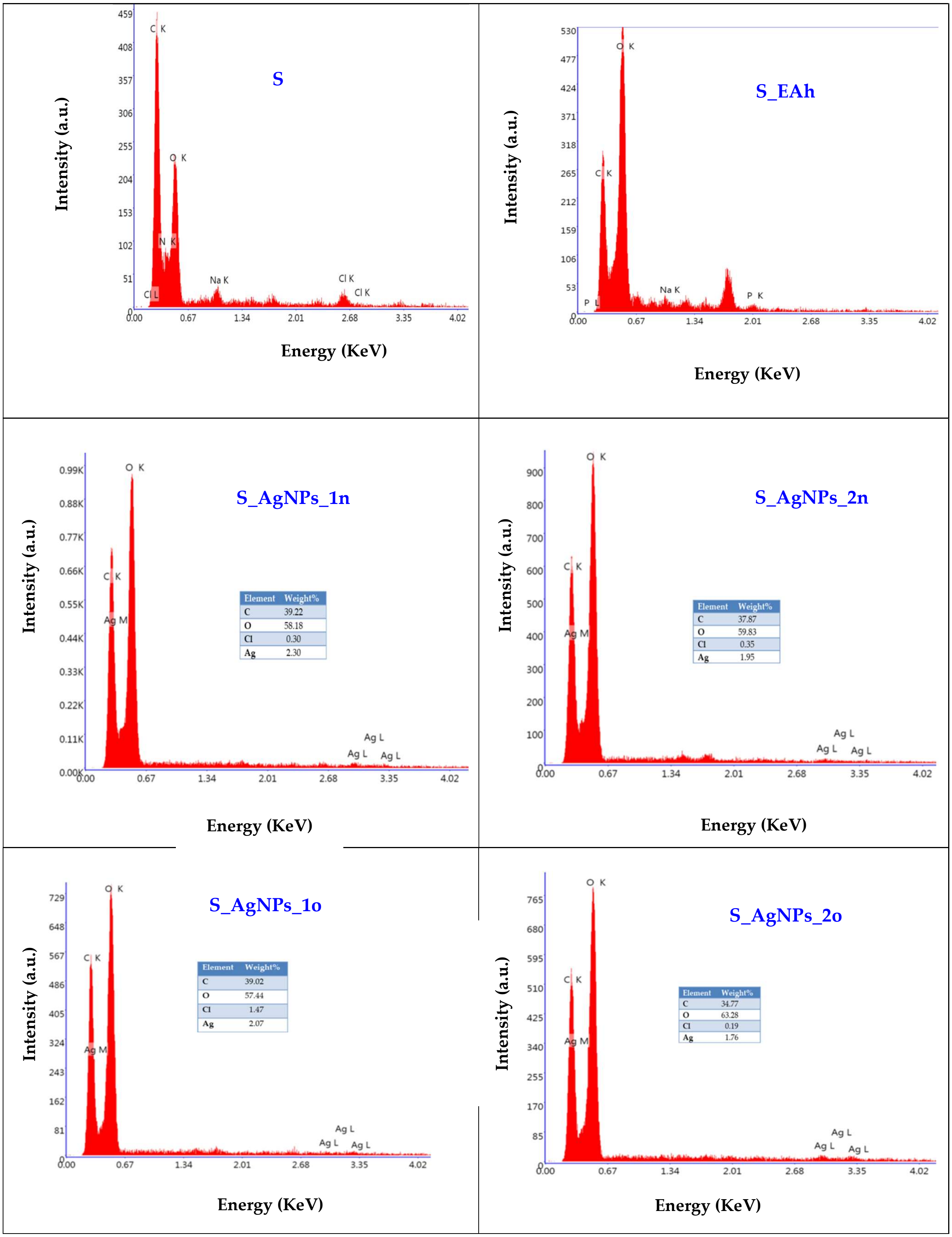

Figure S1. The EDX spectra of all samples: *Andropogon halepensis* extract (EAh) and *Andropogon*-derived AgNPs (AgNPs_1n, AgNPs_1o, AgNPs_2n & AgNPs_2o), spider silk (S) and spider silk composites with plant extract (S_EAh) or with silver nanoparticles (S_AgNPs_1n, S_AgNPs_1o, S_AgNPs_2n & S_AgNPs_2o), at 10 kV.

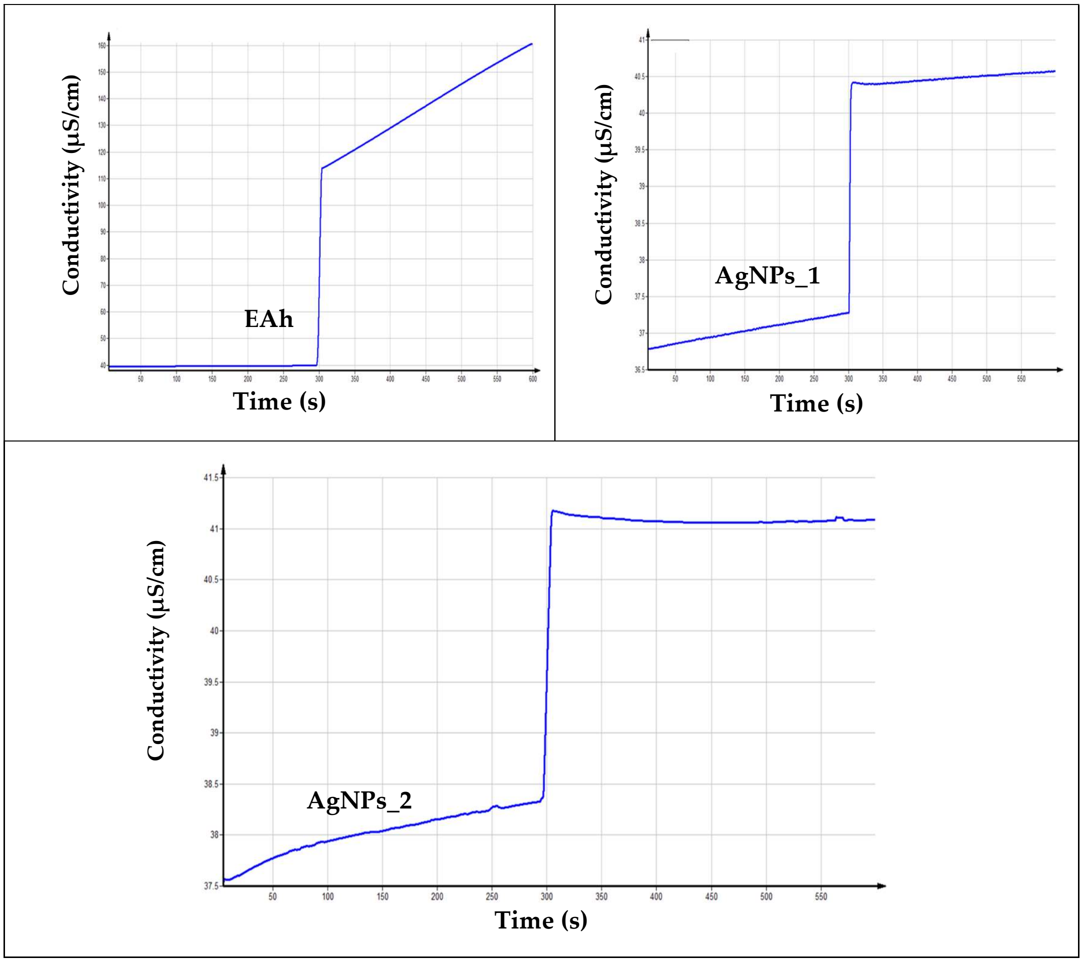

Figure S2. Investigation of urease inhibitory action of *the Andropogon halepensis* extract and of the developed phytometallic nanoparticles, estimated by the conductometric method.

References

1. Barbinta-Patrascu, M.-E.; Chilom, C.; Nichita, C.; Zgura, I.; Iftimie, S.; Antohe, S. Biophysical insights on Jack bean urease in the presence of silver chloride phytonanoparticles generated from *Mentha piperita* L. leaves, *Rom.Rep.Phys.*, **2022**, *74*, 605.
2. Coates, J. Interpretation of Infrared Spectra, a Practical Approach. In Encyclopedia of Analytical Chemistry; Meyers, R.A., Eds.; John Wiley & Sons Ltd: Chichester, UK, 2000, pp. 1-23.
3. Kiseleva, A.; Nestor, G.; Östman, J. R.; Kriuchkova, A.; Savin, A.; Krivoshapkin, P.; Krivoshapkina, E.; Seisenbaeva, G. A.; Kessler, V. G. Modulating Surface Properties of the *Linothele fallax* Spider Web by Solvent Treatment, *Biomacromolecules*, **2021**, *22*, 4945-4955. https://doi.org/10.1021/acs.biomac.1c00787
4. Goto, H.; Kikuchi, R.; Wang, A. Spider Silk/Polyaniline Composite Wire, *Fibers*, **2016**, *4(2)*, 12. https://doi.org/10.3390/fib4020012
5. Gorinstein, S.; Park, Y.S.; Heo, B.G.; Namiesnik, J.; Leontowicz, H.; Leontowicz, M.; Ham, K.S.; Cho, J.Y.; Kang, S.G. A comparative study of phenolic compounds and antioxidant and antiproliferative activities in frequently consumed raw vegetables, *Eur. Food Res. Technol.*, **2009**, *228*, 903-911. https://doi.org/10.1007/s00217- 008-1003-y.
6. Barbinta-Patrascu, M.-E.; Badea, N.; Bacalum, M.; Ungureanu, C.; Suica-Bunghez, I. R., Iordache, S. M.; Pirvu, C.; Zgura, I.; Maraloiu, V.A. 3D hybrid structures based on biomimetic membranes and *Caryophyllus aromaticus* - "green" synthesized nano-silver with improved bioperformances, *Materials Science and Engineering: C*, **2019**, *101*, 120-137. DOI: 10.1016/j.msec.2019.03.069.

7. Seo, S.J.; Das, G.; Shin, H.S.; Patra, J.K. Silk Sericin Protein Materials: Characteristics and Applications in Food-Sector Industries, *Int. J. Mol. Sci.*, **2023**, *24*, 4951. https://doi.org/10.3390/ijms24054951.
8. Barbinta-Patrascu, M.-E.; Nichita, C.; Badea, N.; Ungureanu, C.; Bacalum, M.; Zgura, I.; Iosif, L.; Antohe, S. Biophysical aspects of bio-nanosilver generated from *Urtica dioica* Leaves and *Vitis vinifera* fruits' extracts, *Rom. Rep. Phys.* **2021**, *73*, 601.
9. Shameli, K.; Ahmad, M. B.; Jazayeri, S. D.; Shabanzadeh, P.; Sangpour, P.; Jahangirian, H.; Gharayebi, Y. Investigation of antibacterial properties silver nanoparticles prepared via green method, *Chemistry Central Journal,* **2012**, *6*, 73. doi:10.1186/1752-153X-6-73.

Disclaimer/Publisher's Note: The statements, opinions and data contained in all publications are solely those of the individual author(s) and contributor(s) and not of MDPI and/or the editor(s). MDPI and/or the editor(s) disclaim responsibility for any injury to people or property resulting from any ideas, methods, instructions or products referred to in the content.
